# Seropositive rheumatoid arthritis with concomitant MRI confirmed sacroiliitis in an 18-year-old Saudi female: A case report of diagnostic and therapeutic challenges

**DOI:** 10.1097/MD.0000000000048773

**Published:** 2026-05-15

**Authors:** Fahidah Alenzi, Shahad AlYamini

**Affiliations:** a Department of Internal Medicine, College of Medicine, Princess Nourah bint Abdulrahman University, Riyadh, Saudi Arabia.

**Keywords:** anti-cyclic citrullinated peptide (anti-CCP), axial spondyloarthritis, rheumatoid arthritis, sacroiliitis

## Abstract

**Rationale:**

Rheumatoid arthritis (RA) and axial spondyloarthritis (axSpA) are distinct inflammatory rheumatic diseases with different clinical, serological, and genetic profiles. RA is typically characterized by symmetric peripheral polyarthritis and autoantibody positivity, particularly anti-cyclic citrullinated peptide (anti-CCP), whereas axSpA primarily involves the axial skeleton and sacroiliac joints and is often associated with human leukocyte antigen-B27 (HLA-B27). The coexistence of seropositive RA and axSpA is uncommon and presents diagnostic and therapeutic challenges, particularly when axial symptoms are under-recognized in young patients. This case highlights a rare overlap phenotype and emphasizes the role of advanced imaging and individualized management.

**Patient concerns:**

An 18-year-old Saudi woman presented with inflammatory peripheral joint pain, associated with chronic axial symptoms suggestive of sacroiliac involvement.

**Diagnoses:**

Laboratory investigations revealed markedly elevated anti-cyclic citrullinated peptide (anti-CCP) antibodies (373 U/mL), supporting the diagnosis of seropositive RA based on the 2010 American College of Rheumatology/European Alliance of Associations for Rheumatology criteria. Magnetic resonance imaging of the sacroiliac joints demonstrated bilateral sacroiliitis with active inflammatory changes, fulfilling the Assessment of SpondyloArthritis International Society (ASAS) criteria for axial spondyloarthritis, despite negative HLA-B27 and absence of other typical spondyloarthritis features.

**Interventions:**

The patient was initially managed with methotrexate (15 mg/wk) and low-dose prednisone (5 mg/d). Due to persistent axial symptoms, treatment was escalated to adalimumab following appropriate screening and counseling.

**Outcomes:**

At 3-month follow-up, the patient demonstrated significant clinical improvement (>70%) in both peripheral and axial symptoms, with normalization of inflammatory markers C-reactive protein (CRP) and erythrocyte sedimentation rate.

**Lessons:**

This case underscores the importance of considering overlapping rheumatologic conditions in patients with atypical presentations. Magnetic resonance imaging plays a critical role in detecting axial involvement, particularly in HLA-B27–negative patients. Early recognition of such overlap syndromes is essential to guide appropriate targeted therapy and improve long-term outcomes, especially in young populations.

## 1. Introduction

Rheumatoid arthritis (RA) is a systemic autoimmune disorder characterized by symmetric polyarthritis of the small joints, morning stiffness, and seropositivity for rheumatoid factor (RF) and/or anti-cyclic citrullinated peptide (anti-CCP) antibodies, which have a specificity exceeding 95% for RA.^[1,2]^ In contrast, sacroiliitis is a hallmark of axial spondyloarthritis (axSpA), typically presenting with inflammatory back pain, human leukocyte antigen-B27 (HLA-B27) positivity, and magnetic resonance imaging (MRI) evidence of active sacroiliac (SI) joint inflammation.^[2,3]^ Radiographic sacroiliitis has been reported in up to 20% of patients with RA^[4]^; however, with advancements in imaging and improved classification criteria, many of these cases are now considered misdiagnoses, overlap syndromes, or coincidental comorbidities.^[5,6]^ The true coexistence of RA and axSpA remains rare, with fewer than 15 reported cases worldwide since 2010.^[1,5,7]^ We present a diagnostically complex case of an 18-year-old Saudi female with seropositive RA and MRI confirmed sacroiliitis, highlighting the challenges in disease phenotyping, classification, and management in young patients, particularly in regions with a high prevalence of both RA and spondyloarthritis.^[8]^

## 2. Case report

**Figure 1.**
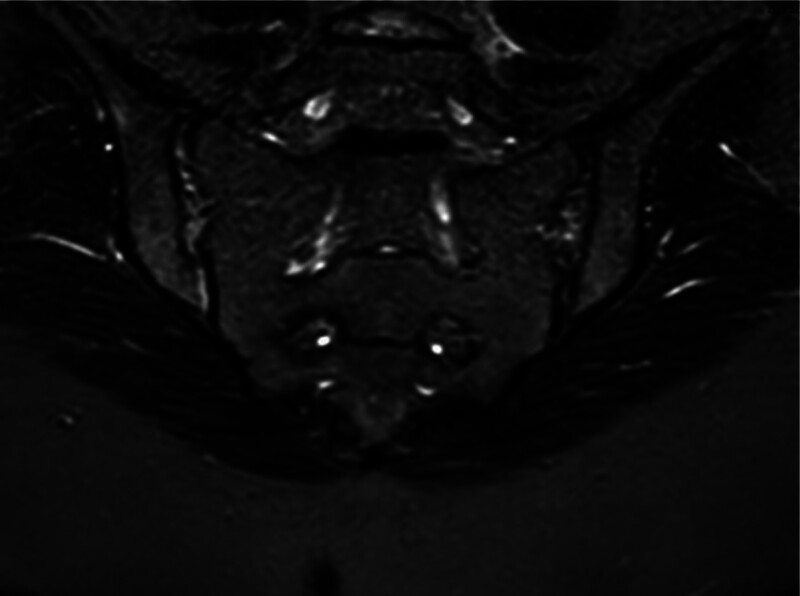
Axial fat-suppressed T2-weighted MRI of the sacroiliac joints showing subchondral bone marrow edema, compatible with active sacroiliitis. MRI = magnetic resonance imaging.

**Figure 2.**
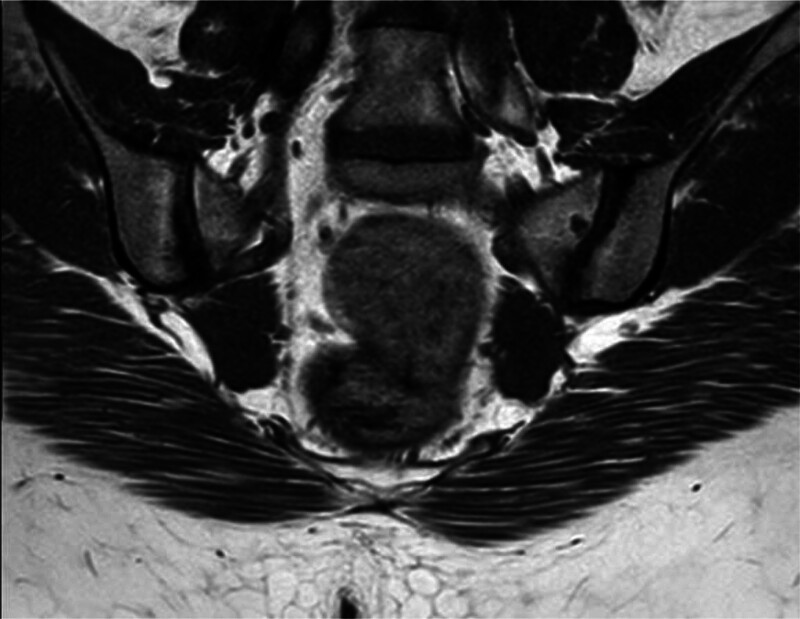
Axial T1-weighted MRI of the sacroiliac joints demonstrates bilateral subchondral sclerosis, with asymmetric fatty metaplasia more pronounced on the right, and a small right-sided subchondral cyst, consistent with chronic structural changes of sacroiliitis. MRI = magnetic resonance imaging.

**Figure 3.**
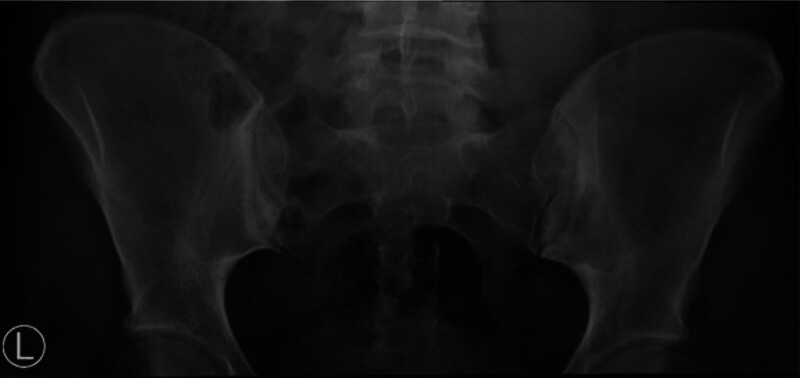
Anteroposterior radiograph of the pelvis demonstrating bilateral sacroiliac joint sclerosis with irregular joint margins and joint space narrowing, findings consistent with chronic structural sacroiliitis grade 2. No complete ankylosis was observed.

An 18-year-old Saudi woman presented with a 6-month history of symmetric small joint pain, morning stiffness lasting more than 1 hour, and intermittent chronic inflammatory low back pain. She denied psoriasis, uveitis, enthesitis, or a family history of spondyloarthritis. On examination, there was tenderness in 14 joints (8 metacarpophalangeal [MCP] and 6 proximal interphalangeal [PIP] joints), with no swelling or deformities. Sacroiliac joint compression reproduced pain, while Schober’s test was normal. Laboratory investigations revealed markedly elevated anti-cyclic citrullinated peptide (anti-CCP) antibodies (373 U/mL; reference < 20 U/mL), negative RF, elevated C-reactive protein (CRP; 12 mg/L; normal < 5 mg/L), and erythrocyte sedimentation rate (28 mm/h; normal < 20 mm/h). Antinuclear antibody (ANA), extractable nuclear antigen panel, and HLA-B27 were negative. Pelvic radiography showed bilateral sacroiliitis without ankylosis (Figs. 1–3). Magnetic resonance imaging of the sacroiliac joints (STIR and T1 post-contrast) demonstrated bilateral, predominantly symmetric sacroiliitis, characterized by subchondral bone marrow edema and erosions involving both the iliac and sacral aspects, consistent with active inflammatory sacroiliitis according to ASAS criteria^[4]^ (Figs. 1 and 2). Hand radiographs showed no erosions. The patient was diagnosed with seropositive rheumatoid arthritis based on the 2010 American College of Rheumatology/European Alliance of Associations for Rheumatology criteria^[9]^ and axial spondyloarthritis according to ASAS criteria (imaging plus clinical features).^[2]^ Given persistent chronic axial symptoms despite methotrexate (15 mg/wk) and low-dose prednisone (5 mg/d), adalimumab, a tumor necrosis factor inhibitor effective in both RA and axSpA, was initiated following tuberculosis screening and vaccination counseling.

At baseline, the DAS28-CRP indicated high disease activity. At 3-month follow-up, the patient demonstrated **>**70% clinical improvement in both peripheral and axial symptoms, with normalization of CRP and erythrocyte sedimentation rate and a DAS28-CRP consistent with low disease activity.

Non-pharmacological management, including spinal mobility exercises, breathing exercises, and low-impact aerobic activity, was implemented alongside patient education to support functional preservation, reflecting a multidisciplinary and holistic approach to care.

## 3. Discussion

This case represents a rare overlap between seropositive rheumatoid arthritis (RA) and axial spondyloarthritis (axSpA), highlighting important diagnostic and therapeutic challenges. Although sacroiliac involvement has been reported in RA, it is typically nonerosive and not associated with active inflammatory changes on MRI. To further evaluate this atypical presentation, antinuclear antibody (ANA) and extractable nuclear antigen testing were performed to assess for potential coexisting systemic autoimmune diseases that could explain the axial involvement. In contrast, the markedly elevated anti-cyclic citrullinated peptide (anti-CCP) level strongly supports the diagnosis of RA, as false positivity is exceedingly rare in the absence of other autoimmune conditions.^[1,10]^ Conversely, MRI confirmed sacroiliitis is highly suggestive of spondyloarthritis, particularly in young patients presenting with inflammatory back pain^[2]^ (Table 1). Several hypotheses may explain the coexistence of rheumatoid arthritis (RA) and axial spondyloarthritis (axSpA). First, a true dual diagnosis is biologically plausible, given shared non-HLA genetic risk factors despite distinct HLA associations. Second, RA may rarely present with atypical axial involvement, such as asymmetric sacroiliitis with erosive changes; however, the presence of bone marrow edema on MRI indicates active inflammation, which is not typical of degenerative or mechanical causes. Third, seropositive axSpA where anti-cyclic citrullinated peptide (anti-CCP) antibodies are detected in approximately 2% to 5% of patients, particularly women with peripheral arthritis may represent an alternative explanation; however, these cases usually demonstrate significantly lower anti-CCP titers than those observed in our patient.^[6,9,11,12]^ Although sacroiliac involvement has been occasionally reported in rheumatoid arthritis (RA), it is typically nonerosive and not associated with active inflammatory changes on MRI. In contrast, our patient demonstrated bilateral bone marrow edema and erosions on MRI, fulfilling the ASAS imaging criteria for active sacroiliitis. These findings are characteristic of axial spondyloarthritis (axSpA) and are not typical of RA-related sacroiliac involvement, making RA alone insufficient to explain the axial disease. We also considered whether this presentation could represent axSpA with peripheral involvement rather than RA. However, the patient exhibited highly specific serological and clinical features of RA, including a markedly elevated anti-cyclic citrullinated peptide (anti-CCP) level (373 U/mL) and symmetric involvement of small joints (metacarpophalangeal and proximal interphalangeal joints), which are not characteristic of axSpA. Additionally, the absence of typical SpA features such as psoriasis, uveitis, enthesitis, or a family history of spondyloarthritis along with negative HLA-B27 status, further reduces the likelihood of axSpA as a unifying diagnosis. Taken together, the presence of distinct and independently classifiable features fulfilling both the 2010 American College of Rheumatology/EULAR criteria for RA and the ASAS criteria for axSpA supports a dual diagnosis as the most accurate explanation of this patient’s clinical, serological, and imaging profile. Therapeutic responses also differ across disease domains: conventional synthetic disease-modifying antirheumatic drugs, such as methotrexate, are effective for peripheral RA but have limited efficacy in axial SpA, whereas interleukin-17 (IL-17) inhibitors are effective in axSpA but less so in RA. In this context, given persistent axial symptoms and limited response to low-dose prednisone, adalimumab was initiated, resulting in significant clinical improvement.^[13-16]^ Aldahneen et al previously reported the first case of a Saudi woman with concomitant rheumatoid arthritis (RA) and ankylosing spondylitis (AS) from the Gulf region.^[17]^ However, several important distinctions differentiateour case. Their patient was 45 years old with long-standing, established disease, whereas our patient is an 18-year-old presenting early in the disease course a stage at which classification criteria for both RA and axial spondyloarthritis (axSpA) are less well validated. Serologically, their patient was positive for both RF and anti-cyclic citrullinated peptide (anti-CCP), consistent with classical RA, whereas our patient was RF-negative but demonstrated a markedly elevated anti-CCP titer (373 U/mL), still strongly supporting RA but potentially reflecting a distinct immunophenotype. Importantly, Aldahneen et al relied on conventional radiography demonstrating a bamboo spine and structural sacroiliitis, indicative of advanced, irreversible disease. In contrast, our case utilized MRI to identify early inflammatory changes, including bone marrow edema and erosions, allowing for earlier detection and intervention prior to irreversible structural damage.^[17]^ In contrast, pelvic MRI in our patient demonstrated active bone marrow edema and erosions, fulfilling the ASAS imaging criteria for inflammatory sacroiliitis and supporting a diagnosis of axial spondyloarthritis rather than degenerative or mechanical back pain.^[2]^ This distinction is consistent with findings reported by Barczyńska et al., whose case series of 6 patients with RA–AS overlap highlighted predominantly radiographic evidence of advanced structural changes, typically observed in middle-aged individuals after prolonged disease evolution.^[5,6,18]^ Together, these findings highlight that not all cases of rheumatoid arthritis with back pain represent the same clinical entity. Our case expands the phenotypic spectrum of RA–SpA overlap to include young, RF-negative patients with markedly elevated anti-CCP levels and early, MRI confirmed inflammatory sacroiliitis, a presentation not previously reported in the Middle East. From a regional perspective, Saudi Arabia and the Gulf region have a substantial burden of both rheumatoid arthritis (RA; prevalence ~0.64%) and spondyloarthritis (SpA; 0.49%)^[19,20]^ (Table 2).^[21-25]^ However, RA with sacroiliitis has rarely been reported in Gulf populations. Given the high rates of consanguinity and unique HLA profiles in this region, such overlap syndromes may be underrecognized. Therefore, MRI should be strongly considered in young seropositive patients with persistent back pain, even in the absence of HLA-B27 or classic SpA features.

**Table 1:** Differentiating RA, axSpA, and overlap features.

Feature	RA	axSpA	Overlap consideration
Age at onset	30–50 yr	<40 yr	Young onset favors axSpA
Sex	♀ > ♂	♂ ≥ ♀	♀ more common in overlap
Autoantibodies	Anti-CCP+, RF+	Typically negative	High-titer anti-CCP supports RA
Joint pattern	Symmetric MCP/PIP	Axial + asymmetric peripheral	Both patterns may coexist
Sacroiliitis	Rare with non-inflammatory	Hallmark, inflammatory	MRI essential
HLA-B27	Negative	Positive in 80–90%	Often negative in overlap
First-line therapy	csDMARDs (MTX)	TNFi/IL-IL7i	TNFi preferred for dual coverage

Anti-CCP+ = anti-cyclic citrullinated peptide antibodies, axSpA = axial spondyloarthritis, csDMARDs = conventional synthetic disease-modifying antirheumatic drug, HLA-B27 = human leukocyte antigen-B27, IL-IL7i = interleukin 17 inhibitor, MRI = magnetic resonance imaging, MTX = methotrexate, RA = rheumatoid arthritis, RF = rheumatoid factor, TNFi = tumor necrosis factor inhibitor.

**Table 2:** Published cases of coexisting rheumatoid arthritis and axial spondyloarthritis or sacroiliitis between 2010 and 2025.

Author-country-year	Journal	Study type	Key feature	Imaging finding	Serology	Final diagnosis
Guo et al (China)-2011^[21]^ Baksay	Chin Med J	Case report	30-year-old woman; peripheral arthritis + back pain	CT/X-ray: bony ankylosing in sacroiliac bone	RF+, Anti-CCP+	RA and AS
Valderilio Feijó Azevedo (Brazil)-2013^[22]^	Braz J Rheumatol	Case report + literature review	65-years-old male; peripheral arthritis + back pain	X-ray: bilateral sacroiliitis (grade 2); syndesmophytes at L4 and L5	RF+, Anti-CCP+	RA and AS
Koca et al (Turkey)-2014^[23]^	Turk J Phys Med Rehab	Case report	38-years-old woman; peripheral arthritis + back pain	X-ray: bilateral sacroiliitis (grade 2)	RF + Anti-CCP+	RA and AS
Barczyńska et al. (Poland)-2015^[5]^	Reumatologia	Case series (n = 3) + literature review	Adults; symmetric polyarthritis + axial stiffness	X-ray: bilateral sacroiliitis (grade 2–4); changes typical for RA	All RF IgM+, (1/3 Anti-CCP+)	RA and AS
Zhang et al (China)-2021^[7]^	Medicine open	Retrospective cohort (n = 22)	Adults; Symmetric, peripheral, and axial arthritis	X-ray/CT: bilateral sacroiliitis (17/22 grade 3)	RF + Anti-CCP+	RA and AS
Flores-Robles et al (Spain)-2022^[24]^	Case Rep Rheumato	Case series (n = 7) + literature review	Adults; back pain (5/7) + asymmetric peripheral arthritis	X-ray/MRI/CT; sacroiliitis, Syndesmophytes (4/7) + peripheral arthritis	RF + Anti-CCP+	RA and AS
Aldahneen et al (Saudi Arabia)-2022^[17]^	Ann Rheumatol Autoimmun	Case report	69-year-old Saudi man; RA + chronic back pain	X-ray/MRI: bilateral sacroiliitis; bamboo spine	RF+, Anti-CCP+	RA and AS
Yilmaz et al (Turkey)-2025^[25]^	Arab J Rheumatol	Case report	A 57-year-old woman RA + chronic back pain	X-ray/MRI: sacroiliitis, mild bone edema	RF+, Anti-CCP+	r-axSpA and RA

Anti-CCP = anti-cyclic citrullinated peptide (antibody), AS = ankylosing spondylitis, axSpA = axial spondyloarthritis, CT = computed tomography, MRI = magnetic resonance imaging, RA = rheumatoid arthritis, RF = rheumatoid factor.

## 4. Conclusion

This case illustrates that seropositive RA and axSpA can coexist especially in young women and underscores that anti-CCP positivity should not rule out axSpA. MRI is critical for confirming inflammatory sacroiliitis, and treatment with biologics effective for both conditions is key to preventing disability. Greater awareness among rheumatologists in high-prevalence regions like the Middle East is vital for timely diagnosis and improved outcomes.

## Acknowledgments

The authors gratefully acknowledge the patient for providing written consent.

## Author contributions

**Conceptualization:** Fahidah Alenzi.

**Data curation:** Shahad AlYamini.

**Supervision:** Fahidah Alenzi.

**Validation:** Fahidah Alenzi.

**Writing – original draft:** Fahidah Alenzi, Shahad AlYamini.

**Writing – review & editing:** Fahidah Alenzi.
